# Early childhood development: an imperative for action and measurement at scale

**DOI:** 10.1136/bmjgh-2018-001302

**Published:** 2019-06-24

**Authors:** Linda Richter, Maureen Black, Pia Britto, Bernadette Daelmans, Chris Desmond, Amanda Devercelli, Tarun Dua, Günther Fink, Jody Heymann, Joan Lombardi, Chunling Lu, Sara Naicker, Emily Vargas-Barón

**Affiliations:** 1 Centre of Excellence in Human Development, University of the Witwatersrand, Johannesburg-Braamfontein, South Africa; 2 RTI International, Research Triangle Park, North Carolina, USA; 3 Early Childhood Development, Unicef USA, New York City, New York, USA; 4 Department of Maternal, Newborn, Child and Adolescent Health, WHO, Geneva, Switzerland; 5 DST-NRF Centre of Excellence in Human Development, University of the Witwatersrand, Johannesburg, South Africa; 6 Early Childhood Development, World Bank Group, Washington, District of Columbia, USA; 7 Department of Mental Health and Substance Abuse, WHO, Geneva, Switzerland; 8 Household Economics and Health Systems, Swiss Tropical and Public Health Institute, Basel, Switzerland; 9 Global Health and Population, Harvard University T H Chan School of Public Health, Boston, Massachusetts, USA; 10 Fielding School of Public Health and WORLD Policy Analysis Center, University of California, Los Angeles, California, USA; 11 Early Opportunities, Washington, District of Columbia, USA; 12 Division of Global Health, Brigham and Women's Hospital and Department of Global Health and Social Medicine, Harvard Medical School, Boston, Massachusetts, USA; 13 RISE Institute, Washington, District of Columbia, USA

**Keywords:** early childhood development, measurement, accountability, sustainable development goals, countdown to 2030, country profiles

## Abstract

Experiences during early childhood shape biological and psychological structures and functions in ways that affect health, well-being and productivity throughout the life course. The science of early childhood and its long-term consequences have generated political momentum to improve early childhood development and elevated action to country, regional and global levels. These advances have made it urgent that a framework, measurement tools and indicators to monitor progress globally and in countries are developed and sustained. We review progress in three areas of measurement contributing to these goals: the development of an index to allow country comparisons of young children’s development that can easily be incorporated into ongoing national surveys; improvements in population-level assessments of young children at risk of poor early development; and the production of country profiles of determinants, drivers and coverage for early childhood development and services using currently available data in 91 countries. While advances in these three areas are encouraging, more investment is needed to standardise measurement tools, regularly collect country data at the population level, and improve country capacity to collect, interpret and use data relevant to monitoring progress in early childhood development.

**Summary box**

- New knowledge of the extent to which experiences during early childhood shape health, well-being and productivity throughout the life course has prompted action to improve early childhood development at the country, regional and global levels.
- Advances have been made in three areas of measurement needed to achieve these goals: population-level child assessments, population proxies of children at risk of poor childhood development, and country and regional profiles of drivers and supports for early childhood development.
- Regular, country-comparable, population-level measurements of childhood development, as well as threats to development and available supports and services, are needed to drive progress and accountability in efforts to improve early childhood development.

## Introduction

Scientific findings from diverse disciplines are in agreement that critical elements of lifelong health, well-being and productivity are shaped during the first 2–3 years of life,^1^ beginning with parental health and well-being.^2^ The experiences and exposures of young children during this time-bound period of neuroplasticity shape the development of both biological and psychological structures and functions across the life course.

Adversities during pregnancy and early childhood, due to undernutrition, stress, poverty, violence, chronic illnesses and exposure to toxins, among others, can disrupt brain development, with consequences that endure throughout life and into future generations.^3 4^ Children whose early development is compromised have fewer personal and social skills and less capacity to benefit from schooling. These deficits limit their work opportunities and earnings as adults.^5^ A corollary of early susceptibility to adversity includes responsiveness to opportunities during these early years. As a result, interventions during the first 3 years of life are more effective and less costly than later efforts to compensate for early adversities and to promote human development.^6^

It is estimated that, in 2010, at least 249 million (43%) children under the age of 5 years in low-income and middle-income countries (LMICs) were at risk of poor early childhood development (ECD) as a consequence of being stunted or living in extreme poverty.^7^ This loss of potential is costly for individuals and societies. The average percentage loss of adult income per year is estimated at 26%, increasing the likelihood of persistent poverty for these children, families and societies.^5^ Assuming 125 million children are born each year with a global average of poor infant growth,^8^ the estimated annual global income loss is US$177 billion.^9^ These impacts have serious consequences on economic growth. Recent World Bank estimates suggest that the average country’s per capital gross domestic product would be 7% higher than it is now had stunting been eliminated when today’s workers were children.^10^ At the global level, human capital accounts for as much as two-thirds of the wealth differences between countries. ECD is the foundation of human capital.^11^

Supported by a growing body of evidence and increasing global interest in this field, ECD is included in the 2015 United Nations Sustainable Development Goals (SDGs). Target 4.2 is ‘improved access to quality early childhood development, care and pre-primary education’. Progress towards achieving this target is measured by indicator 4.2.1, ‘the proportion of children under 5 years of age who are developmentally on track in health, learning and psychosocial well-being, by sex’. ECD is closely linked to other SDGs as well, for example, eradicate poverty (1), end hunger and improve nutrition (2), ensure healthy lives (3), achieve gender equality (5), reduce inequality in and among countries (10), and promote peaceful societies (16), and it is implied in several more.^5^

The United Nations Global Strategy for Women’s, Children’s and Adolescents’ Health, 2016–2030 synthesises the 17 SDGs in three strategies: survive, thrive and transform. Survive refers to sustained and increased reductions in preventable deaths of women, newborns, children and adolescents, as well as stillbirths; thrive refers to children receiving the nurturing care necessary to reach their developmental potential; and transform refers to comprehensive changes in policies, programmes and services for women, children and adolescents to achieve their potential.^12^

ECD has also become an important component of other global agendas, including Scaling Up Nutrition, the Global Partnership for Education, the Global Financing Facility for Every Woman Every Child, the Every Woman Every Child movement, the work plans of the WHO, Unicef and the World Bank Group, the G20,^13^ international funding agencies, and philanthropic foundations.^7^

These multifaceted findings have generated political momentum to improve ECD as a critical phase in the life course, making it urgent to develop measurement tools and indicators to monitor progress globally and in countries. Advances in measurement are needed to support efforts to motivate and track political and financial commitments, and to monitor implementation and impact. This means that we must be able to determine how many and which children are thriving, and on track in health, learning and psychosocial well-being.

Measurement of children’s progress in childhood is acknowledged to be challenging because development is by nature dynamic and children have varying individual trajectories. Well-validated instruments of individual development are complex and require extensive training and expertise. These challenges are amplified in efforts to make measurements across populations of children. Taking these limitations into account, we review progress in three areas of measurement that are contributing data to the current political momentum for ECD and efforts to monitor implementation and impact. Progress is being made to construct a feasible country-comparable measure of young children’s development that could be incorporated into national surveys, to improve proxies of population levels of young children at risk of poor early development, and to generate country profiles of determinants, drivers and coverage for early childhood development and services, using currently available data.

## A new initiative to construct a population measure of ECD

A direct measure of the development of children 0–5 years that could be administered globally and used both within and across countries is urgently needed. Efforts have been made since the 1980s to develop a globally applicable measure of ECD, with the major challenges being individual and cultural variations in the onset of early skills.^14^

Currently, the Early Child Development Index (ECDI) is included as the indicator of SDG goal 4, target 4.2. It is a composite index, first introduced in Unicef’s fourth Multiple Indicator Cluster Survey (MICS) in 2010. It is derived from 10 caregiver-reported questions designed for children aged 36–59 months to assess four domains of development: literacy-numeracy, learning/cognition, physical development and socioemotional development. Some items are acknowledged to be unsuitable for assessing development,^15^ and efforts are under way to revise the index, as well as to include items applicable to children younger than 3 years of age.

Three research efforts have collaborated to create the *Global Scale for Early Development* (GSED): the Infant and Young Child Development from the WHO,^16^ the Caregiver-Reported Early Development Instrument from the Harvard Graduate School of Education,^17^ and the Developmental Score from the Global Child Development Group at the University of the West Indies.^18^ The goals of the GSED are to develop two instruments for measuring ECD (0–3 years) globally: a population-based instrument and a programme evaluation instrument, as described in table 1.

**Table 1:** Global Scale for Early Development: population and programme measures

	Goal	Administration	Estimated duration (min)	Score
Population	Map childhood development status globally. Track trajectories of childhood development over time. Monitor benefits of population-level interventions. Draw attention to populations in need of support, including monitoring impact of humanitarian emergencies and other crises.	Caregiver report.	5–10	Holistic.
Programme	Identify populations of children at risk of poor developmental trajectories. Quantify the impact of an intervention on developmental outcomes.	Caregiver report combined with direct assessment.	<30	Domains of development (motor, cognitive, language and so on).

The GSED takes advantage of large-scale and cohort studies from many countries and is harmonising efforts to generate population-based and programmatic evaluation measures of the development of children aged 0–3 years old that can be used globally (table 2). The scale will be available for country testing in 2019. The aim is to have the population-based measure incorporated into national surveys, including Unicef’s MICS and the US Agency for International Development’s Demographic and Health Surveys (DHS), to produce globally comparable monitoring data. Efforts are also under way to harmonise the revision of ECDI and the development of GSED to align on child outcome measurement from birth to 59 months of age.

**Table 2:** Development and validation of the Global Scale for Early Development

Predefined characteristics	Methodology for prototype creation	Validation
Culturally neutral, minimal adaptation required. Psychometrically sound, including concurrent, discriminant and predictive validity. Sensitive to child age and environment. Amenable to improvements. Feasibly administered. Minimal training required. Interpretable by policy makers. Open access, no/limited cost (training, materials). Could be used to develop norms.	Creation of a common data set from three initiatives (IYCD, CREDI and D-score). Harmonisation of data sets generated from the use of 22 instruments (2275 items) administered to 73 222 children (a total of 109 079 visits, and unique child/age combinations) within 51 cohorts. Items selected by matching ratings of subject matter experts with instrument prototypes based on construction of a latent scale of childhood development using optimal statistical modelling approaches (Rasch vs a two-parameter model).	Simulations of expected psychometric properties of the instruments based on existing data set. Field testing to verify the properties of both instruments, in accordance with the predefined characteristics.

CREDI, Caregiver-Reported Early Development Instrument; D-score, Developmental Score; IYCD, Infant and Young Child Development.

## A country-comparable proxy for population levels of risk of poor childhood development

Information about children’s risk for poor development is important, as is identifying areas for intervention. To track these, a proxy measure of population levels of young children at risk of suboptimal development has been calculated.

Stunting and poverty were used in the first published estimation in 2007 of the global prevalence of risk to children’s development. The initial choice of indicators was based on evidence that they both predict poor cognitive development and school performance.^19 20^ Additional advantages are that their definitions are standardised and many countries have data on both indicators.^21^

Lu *et al* 21 updated the earlier values to 2010, using the 2006 WHO growth standards and World Bank poverty rates (US$1.25 per person per day), leading to an estimate of 249 million children or 43% of all children under 5 years of age in LMICs being at risk of poor childhood development. The accuracy and comparability of the later estimates benefited greatly from major advances in both data availability and estimation methods.^21^

To estimate the long-term consequences of poor ECD, studies focus on estimating the impact on subsequent schooling and labour market participation and wages. The current estimate, that the average percentage of annual adult income lost as a result of stunting and extreme poverty in early childhood is about 26%, is supported by follow-up adult data from early life interventions. Two programmes have found wage increases between 25% in Jamaica attributed to a psychosocial intervention^22^ and 46% in Guatemala attributed to a protein supplement.^23^

In order to improve the estimate of risk, efforts are under way to include additional risks experienced in ECD known to affect health and well-being across the life course. For example, adding low maternal schooling and exposure to harsh punishment to stunting and extreme poverty, for 15 countries with available data from MICS in 2010/2011, increased the number of children estimated to be at risk of poor childhood development substantially.^5^

## Country profiles of ECD

Population-based measures of early child development and proxies of children at risk give an indication of prevalence, and indicators of disparity can be derived according to gender, urban–rural location and socioeconomic status. However, they do not include drivers, determinants nor coverage of interventions that could improve childhood development.

The *Countdown to 2015* *for Maternal, Newborn and Child Survival*, established in 2005, set a precedent by creating mechanisms to portray multidimensional aspects of progress towards improving maternal and child health, and is testimony of its value.^24^ *Countdown to 2030*, which tracks maternal, child and adolescent health and nutrition goals, has expanded to address the broader SDG agenda, including ECD, health in humanitarian settings and conflict, and adolescent health and well-being.^25 26^ It includes coverage and equity of essential interventions, as well as indicators of determinants and the enabling environment provided by policies.

This approach has been applied to ECD using the Nurturing Care Framework,^27^ launched at the 71st World Health Assembly. The concept of nurturing care was introduced in the 2017 *Lancet* *Series* *Advancing Early* *Child* *Development: From Science to Scale*. Nurturing Care Framework comprises conditions for early development: good health and nutrition; protection from environmental and personal harm; affectionate and encouraging responses to young children’s communications; and opportunities for young children to learn through exploration and interpersonal interactions.^7^

These early experiences are nested in caregiver–child and family relationships. In turn, parents, families and other caregivers require support from a facilitating environment of policies, services and communities. Policies, services and programmes can protect women’s health and well-being, safeguard pregnancy and birth, and enable families and caregivers to promote and protect young children’s development.^6^

The Nurturing Care Framework has been used to produce ECD profiles for 91 LMICs.^28^ Countries were selected either to ensure alignment of ECD with *Countdown to 2030*, or because more than 30% of children are estimated to be at risk of poor ECD in 2010, using the methods described in Lu *et al* 21 and Black *et* *al*.^7^

These country profiles, which consist of currently available data from LMICs, are laid out to represent the Nurturing Care Framework. The profiles consist of the following sections:

- Selected demographic indicators of the country relevant to early child development: total population, annual births, children under 5 years of age and under-5 mortality.
- Threats to ECD, including maternal mortality, young motherhood, low birth weight, preterm births, child poverty, under-5 stunting, harsh punishment and inadequate supervision.
- The prevalence of young children at risk of poor child development disaggregated by gender and rural–urban residence, and lifetime costs of growth deficit in early childhood in US dollars.
- The facilitating policy environment for caregivers and children, as indexed by relevant conventions and national policies.
- Support and services to promote ECD in the five areas of nurturing care: early learning, health, nutrition, responsive caregiving, and security and safety.

Most of the existing data are published in Unicef’s annual State of the World’s Children. Convention and policy data come from, among others, the United Nations Treaty Collections and the International Labour Organization.

**Figure 1:**
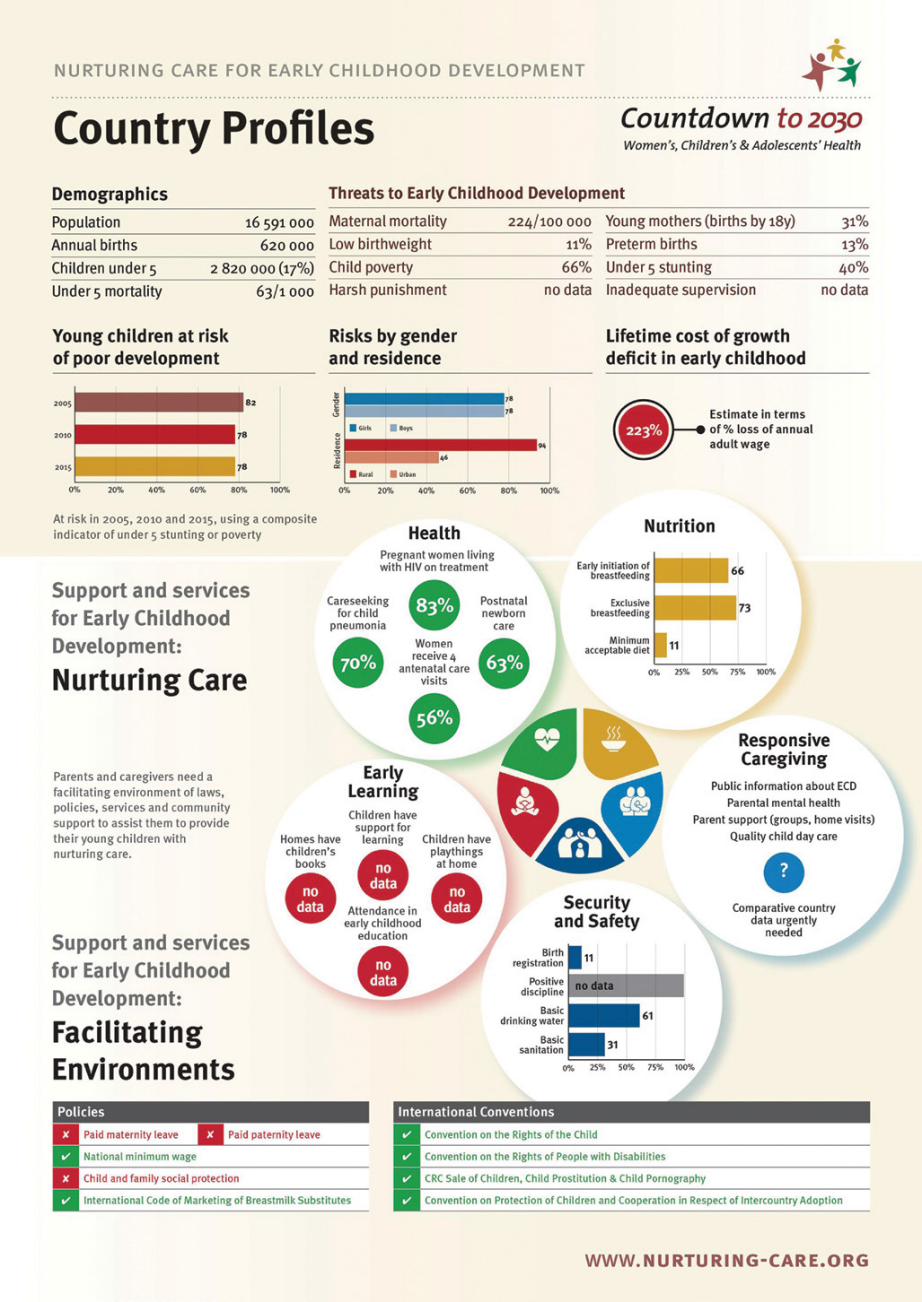
An example of an early childhood development (ECD) country profile. CRC, convention on the rights of the child.

Figure 1 shows an example of the country profiles, with the country name replace by ‘Country Profiles’.

**Figure 2:**
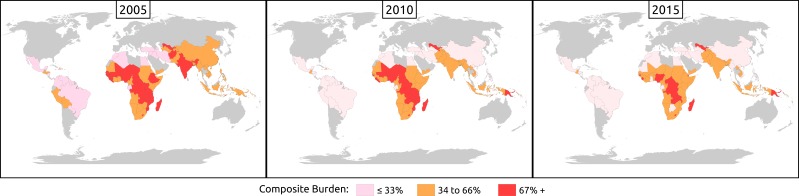
Decline in the number of countries with high proportions of young children at risk of poor development between 2005 and 2015.

**Figure 3:**
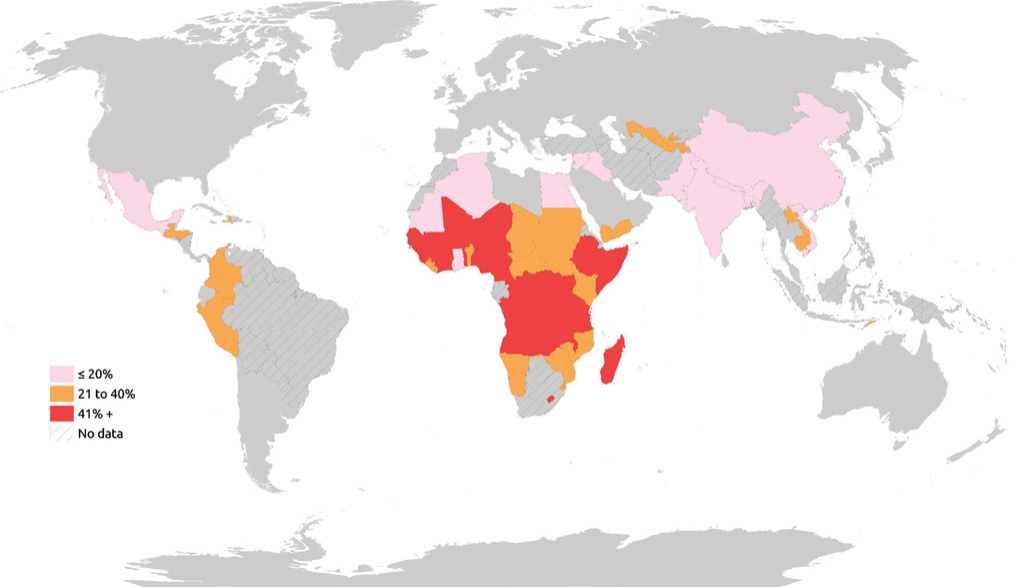
Differences in risk of poor development among urban and rural children in 63 countries (most recent years with available data).

In a forthcoming paper, Lu and Richter (2019) describe in detail the updated estimates of children at risk of poor childhood development using the newly released poverty line of US$1.9 per person per day to estimate that, in 2015, 233 million children or 40.5% of children under 5 years of age were at risk of poor childhood development. Figures 2 and 3 show the estimates of risk for poor ECD across a decade, from 2005 to 2010 and 2015, and using the 2010 data variations between children at risk living in rural and urban areas. Gender is not illustrated here because, in most countries, the differences are small and not statistically significant.

Figure 2 shows that, between 2005 and 2010, countries with two-thirds of young children at risk (>67%) declined in both central Europe and South-East Asia. There was little change in countries with high proportions of young children at risk in sub-Saharan Africa during this period, and by 2015 countries with the highest proportion of children at risk were in Central and Southern Africa.

Estimates on the prevalence of children at risk of poor development in urban and rural areas were derived using DHS, MICS and country data for 63 countries with available data in most recent years (figure 3). The differences are strikingly high, with more rural children at risk than their urban counterparts in 50 countries (differences of more than 20%). Almost all countries with 40% point differences were in sub-Saharan Africa.^28^

There are additional indicators that ideally should be included in a monitoring framework, but currently lack comparable country data. Data are usually unavailable because reliable, valid instruments feasible for multicountry administration are still in development, or the instruments are not yet included in representative surveys. In particular, there are as yet no global population-based indicators for assessing responsive caregiving. Suggestions have been made that data should be collected on whether information about ECD and caregiver–child interaction is publicly disseminated, whether home visits or groups are provided for parents at high risk of experiencing difficulties providing their children with nurturing care, and whether affordable good quality child day care is available for families who need it.^29^ National data on laws and policies that support responsive caregiving are also insufficient, for example, wages and other forms of income to enable families to provide for their young children.^30^

Additional data gaps concern risks arising from poor parental mental health,^31^ low maternal schooling, and maternal tobacco and alcohol use, among others, prevalence of childhood developmental delays and disabilities,^32^ and maltreatment and institutionalisation of young children.^33^ There is also no comparable information on government budget allocation to ECD or household expenditure on ECD services care, among others.

## Conclusion

Multidisciplinary scientific evidence and political momentum are focusing on ECD as a critical phase in enhancing health and well-being across the life course. Additional measurements and indicators for monitoring and evaluation are urgently needed to support expansions in implementation and investment, and to report progress. New data will stimulate global, regional and national action, and in turn motivate for more areas of ECD to be covered in national surveys.

The Nurturing Care Framework provides a platform for three important areas of work. First, very significant progress is being made through the revision of the ECDI and the development of the GSED, a short caregiver-reported population measure of ECD that could feasibly be included in DHS, MICS and other nationally representative household surveys. The GSED will enable ECD to be tracked at population levels, and for programmes and services to be monitored and evaluated in comparable ways.

Second, a country-comparable proxy of the risk of poor ECD developed from 2004 data and updated with 2010 data has been extended to 2015, enabling comparisons to be made globally, regionally and by country across the last decade. Plans are in place to update these estimates regularly, and to add new risks as data for more countries become available.

Third, using these estimates, data included in *Countdown to* *2030*, and additional data from MICS and policy databases, initial profiles have been constructed for 91 LMICs. The profiles are organised according to the ecological model of the Nurturing Care Framework with policies, services and programmes supporting families and caregivers to provide good health and nutrition, security and safety, opportunities for early learning, and responsive caregiving for young children to thrive. The further development of these profiles is overseen by a multiagency committee as part of *Countdown to* *2030* and are freely available (http://www.ecdan.org/countries.html and https://nurturing-care.org/?page_id=703). Unicef will update the country data annually and the profiles will be reproduced every 2 years.

However, as indicated earlier, substantial gaps in national and global data on topics of concern to ECD remain. The current global estimation on burden of risks, for example, does not include known risk factors other than stunting and extreme poverty, as a result of which the existing burden calculation is considerably underestimated.^5^ The limited information on ECD investments at the country and global levels is exacerbated by the lack of appreciation of what constitute essential and continuous services, standard indicators for measuring ECD interventions and policies, as well as systematically collected data. Country capacity needs to be strengthened and ECD costing modules integrated into existing household income or expenditure surveys, and routinely collected from specific types of programmes. Clear definitions are needed to track donor contributions to ECD, and efforts should be made to address data issues, including collecting data from emerging donor countries (eg, China), foundations and international non-governmental organisations that are playing an increasing role in financing ECD, as has been called for by the G20.^33^ National policies, strategic plans and laws which support ECD through nurturing care should be tracked for this intersectoral area.

To improve measurements of risks, intervention coverage, policies, financial commitments and impact on young children’s development, more investment is needed to regularly collect and disseminate data at the national and subnational levels. Analytical gaps at the country and global levels exist, especially with respect to equity analyses by household wealth, maternal education and rural–urban location, as well as by gender and child age within 0–5 years.

In conclusion, progress has been extremely positive, but too slow and too fragmented for the bold global agenda of ECD and the Nurturing Care Framework. The alliance with *Countdown to* *2030* is helpful as there is much to be learnt from the initiative’s experience under the Millennium Development Goals (MDGs), as well as collaboration with the SDGs. The country profiles boldly portray what we currently know about ECD in some of the most at-risk conditions and will prove a valuable tool for advocacy and implementation, including to improve measurement. Successful implementation and impact are dependent on accountability supported by regularly updated reliable and valid information.
